# “Deep” Sequencing Accuracy and Reproducibility Using Roche/454 Technology for Inferring Co-Receptor Usage in HIV-1

**DOI:** 10.1371/journal.pone.0099508

**Published:** 2014-06-24

**Authors:** David J. H. F. Knapp, Rachel A. McGovern, Art F. Y. Poon, Xiaoyin Zhong, Dennison Chan, Luke C. Swenson, Winnie Dong, P. Richard Harrigan

**Affiliations:** 1 BC Centre for Excellence in HIV/AIDS, Vancouver, BC, Canada; 2 Faculty of Medicine, University of British Columbia, Vancouver, BC, Canada; INSERM, France

## Abstract

Next generation, “deep”, sequencing has increasing applications both clinically and in disparate fields of research. This study investigates the accuracy and reproducibility of “deep” sequencing as applied to co-receptor prediction using the V3 loop of Human Immunodeficiency Virus-1. Despite increasing use in HIV co-receptor prediction, the accuracy and reproducibility of deep sequencing technology, and the factors which can affect it, have received only a limited level of investigation. To accomplish this, repeated deep sequencing results were generated using the Roche GS-FLX (454) from a number of sources including a non-homogeneous clinical sample (N = 47 replicates over 18 deep sequencing runs), and a large clinical cohort from the MOTIVATE and A400129 studies (N = 1521). For repeated measurements of a non-homogeneous clinical sample, increasing input copy number both decreased variance in the measured proportion of non-R5 using virus (p<<0.001 and 0.02 for single replicates and triplicates respectively) and increased measured viral diversity (p<0.001; multiple measures). Detection of sequences with a mean abundance less than 1% abundance showed a 2 fold increase in median coefficient of variation (CV) in repeated measurements of a non-homogeneous clinical sample, and a 2.7 fold increase in CV in the MOTIVATE/A400129 dataset compared to sequences with ≥1% abundance. An unexpected source of error included read position, with low accuracy reads occurring more frequently towards the edge of sequencing regions (p<<0.001). Overall, the primary source of variability was sampling error caused by low input copy number/minority species prevalence, though other sources of error including sequence intrinsic, temporal, and read-position related errors were detected.

## Introduction

Next generation, “deep”, sequencing is a powerful sequencing method which allows the interrogation of individual template DNA molecules by physical separation [1]. This method has been applied in many different fields including the analysis of mutations in cancer [2], [3], analysis of clonal dynamics in hematopoietic stem cells [4], [5], and in the study of mutational pathways in various viruses [6] among other applications.

One application has been in the prediction of chemokine co-receptor usage in Human Immunodeficiency Virus Type 1 (HIV) based on sequencing the V3 loop [7]–[12]. HIV infects cells using the human CD4 receptor together with the chemokine receptor CXCR4 and/or CCR5 [13], [14]. Co-receptor usage is referred to as viral tropism, of which there are three possible profiles; CXCR4-using “X4” virus, CCR5-using “R5” virus, as well as “D/M” which can indicate either a viral population able to use both receptors (dual), or a population containing both X4 and R5 virus (mixed) [13], [14]. The use of CCR5 antagonists such as maraviroc (MVC) which selectively block the entry of CCR5-using (but not non-R5 using) virus [15], [16] has made prediction of viral tropism important in treatment decisions in HIV.

Despite its increasingly wide-spread use, 454-based deep sequencing is prone to error, including well-documented biases such as errors introduced by stretches of homopolymer and GC content biases [1], [17]. In addition to these several other sources of error have also been described, including primer related selective amplification and in vitro recombination [18]. While overall deep sequencing has been shown to be fairly reproducible down to ∼1% minority species [17]–[19], these sources of error, together with sampling error caused by low template availability [20], could result in substantial uncertainty for rare variants. Further, studies on the reproducibility of deep sequencing data have included low numbers of samples, with relatively low numbers of replicates. Here we report a large scale analysis of the accuracy and reproducibility of deep sequencing as applied to the detection of non-R5 using HIV.

## Materials and Methods

### Ethical Approval

Patients provided written informed consent as part of the clinical studies as approved by the ethics boards at each study site. Ethical approval for this study was provided by the University of British Columbia/Providence Health Care Research Ethics Board. Short read sequence data from this study are available European Nucleotide Archive with study accession number is: PRJEB6005 (secondary study accession number: ERP005461number).

### Sample materials

First, control beads (Roche, Basel, Switzerland) of known sequence were used to determine error rates and positional biases of the sequencing system. Next, Retroviridae Lentivirus Human Immunodeficiency Virus 1 was used from three distinct sources in this study. First were the laboratory grown viruses of known tropism Bal and pNL4-3 (virus derivation described in [20]). Next, was a clinical sample from British Columbia with high viral load that was known to be CCR5 antagonist-naive. Finally, samples from the MOTIVATE and A400129 studies [16], [21] for which deep sequencing and population sequencing results were available (N = 1521) were also used.

### Sample processing

Viral RNA was extracted from 500 µL of primary patient plasma using the the NucliSens easyMAG, and eluted in 60 µL of NucliSens easyMAG Extraction Buffer 3. Extracts were then amplified in triplicate, unless otherwise stated, via nested RT-PCR using multiplex identifier (MID) tagged primers (reaction conditions and primer sequences described in Methods S1). Following extraction, samples were quantified using the Invitrogen Quant-iT PicoGreen dsDNA Reagent assay on the DTX 880 Multimode Detector (Beckman Coulter, Fullerton, CA, USA). Replicate amplifications combined in equal proportions, purified, re-quantified, and then deep sequenced (details in Methods S1).

### Alignment and scoring

Raw sequencing files were processed using an in-house pipeline described in Methods S1. Following processing, unique sequences exhibiting deletions of 1 or 2 base pairs in length, having a total length less than 99 base pairs, and/or those with internal stop codons were rejected. All remaining unique sequences were run through a Position Specific Scoring Matrix (PSSM) [22]. Each unique sequence was then assigned a PSSM score, from which co-receptor usage could be inferred. A PSSM score less than −4.75 was inferred as R5 virus, whereas a score greater than or equal to −4.75 was inferred as non-R5 virus [23]. Following scoring, any sample with 750 sequencing reads or greater and at least one sequence exhibiting an exact sequence match between the forward and reverse direction were retained in order to allow comparison between forward and reverse directions. (The requirement of a minimum of 750 reads was chosen by comparison of the “% X4” obtained from the forward and reverse reads including all data collected. Where there were less than 750 reads obtained from a given direction, the coefficient of variation (CV) between the forward and reverse reads increased markedly). The prevalence of each sequence was then determined and the percentage of non-R5 variants calculated independently in both the forward and reverse directions each sample. The mean percentage non-R5 virus between the forward and reverse directions was then calculated. This average was used in all subsequent calculations where the two directions were not being directly compared.

### Reproducibility using a non-homogeneous clinical sample

A non-homogeneous clinical sample was diluted in plasma from HIV-seronegative individuals to a viral load of 8580 copies/mL (286 input copies/reaction), extracted once and amplified in triplicate with a single round of RT-PCR. A separate nested PCR step was performed from this using each of the 12 MIDs (sequences in Methods S1). Replicates were then deep sequenced independently over 18 sequencing, resulting in a total of 47 replicate results.

### The effects of input copy number and replication

In order to probe the effect of viral load on assay sensitivity, a serial dilution (into HIV-negative plasma) of this repeated clinical sample was performed, yielding input viral loads of approximately 8580, 4290, 2145, 1072, 536, 268, and 134 copies/mL. Two extractions were performed at each input viral load and each was amplified and sequenced.

To test the effect of replication combined with that of input copy number, dilutions of the repeated clinical sample were made to viral loads 8580 and 536 copies/mL (286 and 18 input copies/reaction). Each dilution was separately extracted and amplified in triplicate using each of the 12 MIDs and deep sequenced. Additionally, a separate single amplification was made and deep sequenced for each MID at each dilution to allow comparison of single and triplicate amplification.

## Results

### Accuracy and reproducibility of deep sequencing

**Figure 1:**
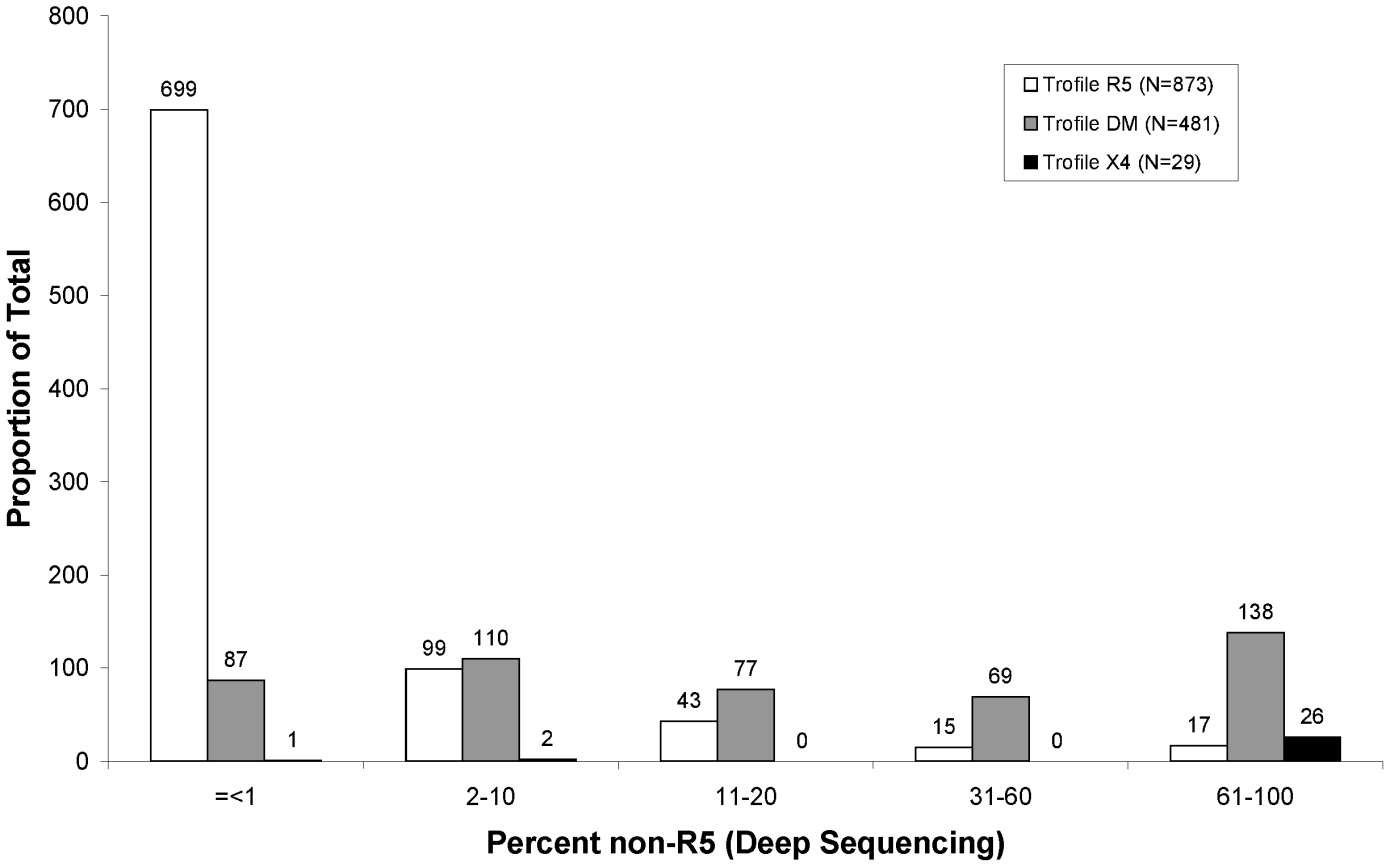
Histogram of percent non-R5 by Trofile call of patients in the MOTIVATE and A4001029 trials for which both population sequencing test results and original Trofile assay results were available (N = 1383). Patients are grouped according to the percent non-R5 measured by deep sequencing and Trofile call. R5 Trofile calls are shown in white. Dual Mixed Trofile calls are shown in grey. X4 Trofile calls are shown in black.

In the subset of the MOTIVATE/A4001029 dataset for which phenotypic measurement of viral tropism was available (N = 1383), the percent non-R5 usage by deep sequencing generally agreed with calls from the original Trofile assay (Monogram Biosciences, San Fransisco, CA, USA) (Fig. 1). Samples called X4 by Trofile (N = 29) had a median of 99.9% (IQR 99.3–100.0) non-R5 virus by deep sequencing, those called D/M (N = 481) had a median of 19.4% (IQR 2.2–73.5) non-R5 virus by deep sequencing, and those called R5 (N = 873) had a median of 0% (IQR 0.0–1.2) non-R5 virus by deep sequencing (Fig. 1). Samples called non-R5 by population-based sequencing (N = 361) had median of 46.0% (IQR 13.0–98.4) non-R5 virus by deep sequencing and those called R5 by population-based sequencing (N = 1022) had median of 0% (IQR 0.0–1.2) non-R5 virus. Concordance of 79.4% was observed between tropism calls by the original Trofile assay and population-based sequencing. In the instance of discordances between tropism calls by the original Trofile assay and population sequencing, a median of 3.1% (IQR 0.2–16.5) non-R5 virus by deep sequencing was observed. While these data suggest that the three assays are generally in accordance, they also show that rare variants are more likely to be missed.

**Figure 2:**
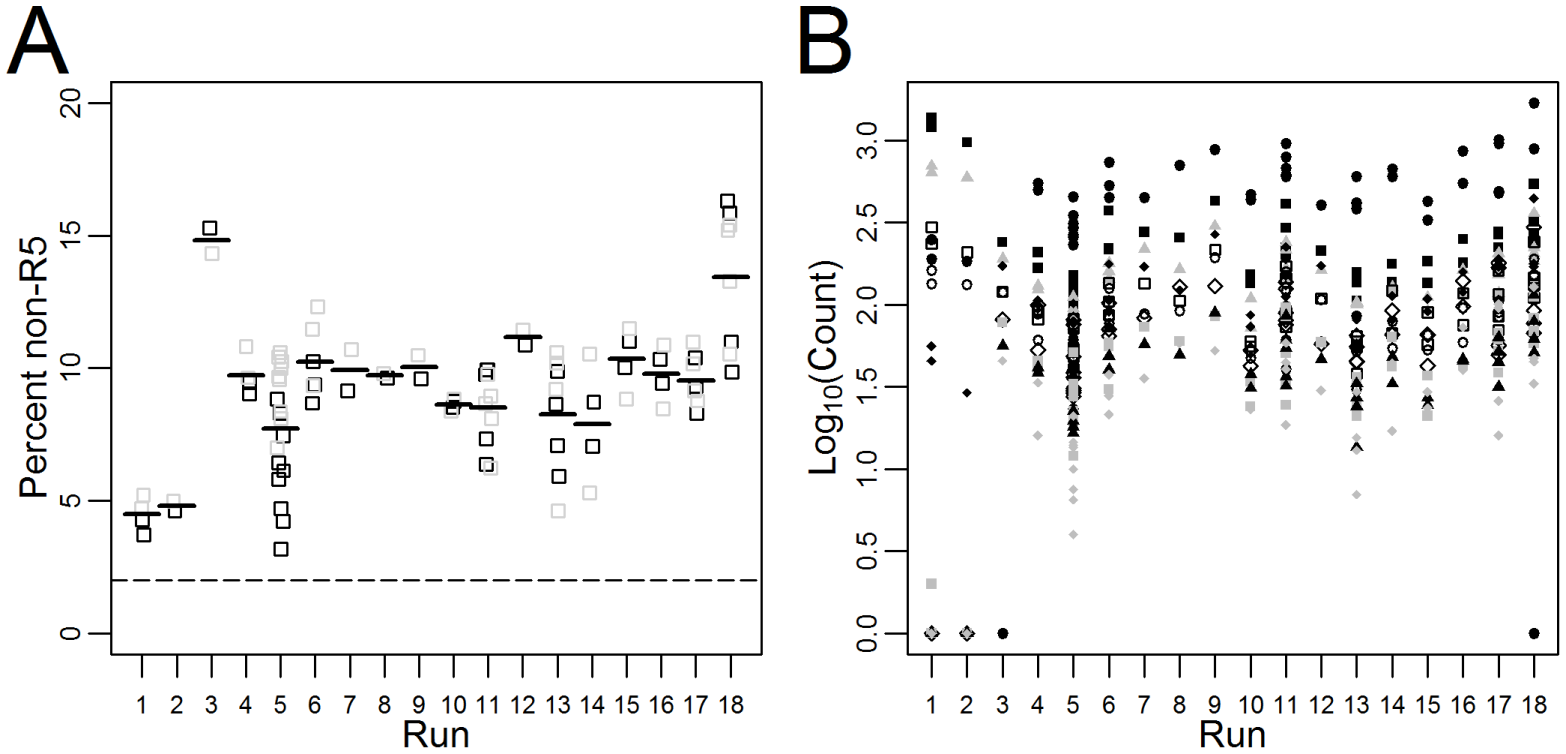
Reproducibility over time by sequence prevalence for a non-homogenous clinical sample (N = 47 replicates over 18 deep sequencing runs). A) The proportion of non-R5 virus (defined as PSSM≥−4.75) measured by sequencing run. Sequences in the “forward” direction are shown in black. Sequences in the “reverse” direction are shown in grey. Thick lines represent the overall mean percentage non-R5 virus measured from all sequences for that run. B) The number of times the 10 most common unique sequences were observed for the non-homogenous clinical sample are plotted against the sequencing run. Each sequence has a unique shade-shape combination.

In order to test the reproducibility of the assay, a non-homogeneous clinical sample was measured a total of 47 times over the course of 18 individual sequencing runs. Some variability in the measured proportion of non-R5 virus was observed between sequencing runs with measurements between 4.0% and 15.3% non-R5 (Fig. 2A), though overall the mean percentage of non-R5 virus measured for each run tended to be close to the overall mean of 9.15% (standard deviation 2.4%) (Fig. 2A). The first 2 runs appeared to have both a low prevalence of non-R5 virus (4.5% and 4.8% non-R5 respectively) (Fig. 2A), as well as differences in the order of most common sequences (Fig. 2B). Despite variability in sequence prevalence, non-R5 virus for the clinical sample was above the 2% cutoff [24] used as a predictor of response to maraviroc for all sequencing runs (Fig. 2A).

### Limits of detection

The ability to detect minority non-R5 virus was tested by spiking varying concentrations of NL4-3 (X4 tropic) into Bal (R5 tropic) to a total viral load of 5000 copies/mL (167 input copies/reaction) (Fig. S1). Measured prevalence of non-R5 virus gave a Spearman's rank correlation Rho of 0.87 (p<<0.001) compared to known prevalence of non-R5 using virus (Fig. S1). Some differences from the known value (based on the amount of NL4-3 spiked into Bal), particularly at low but non-zero proportions of non-R5 virus occurred (Fig. S1). Below 10% prevalence, non-R5 virus was not detected in all instances, particularly below 2–3% where non-R5 virus was rarely detected, though some detection was observed as low as 1% (Fig. S1).

**Figure 3:**
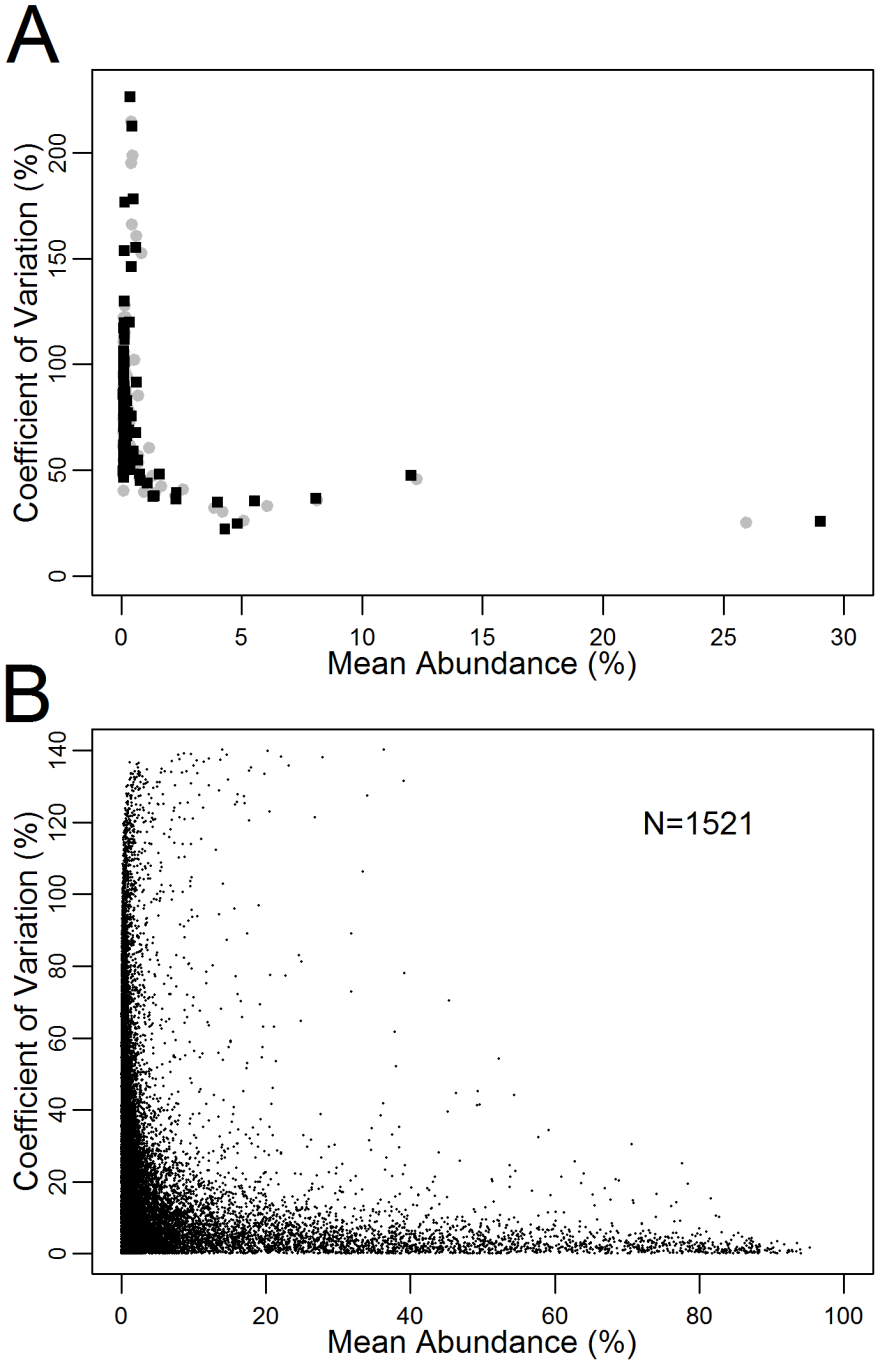
Variability as a function of sequence abundance. The forward direction is indicated in black, the reverse direction in grey. A) Variability in the 100 most common sequences from a non-homogeneous clinical sample run multiple times (N = 47 replicates over 18 deep sequencing runs). B) Variability between the forward and reverse direction for samples in MOTIVATE/A4001029 dataset (N = 1521 samples; 41030 unique sequences, median 26 [IQR 19–34] per sample). Less abundant sequences tended to have more variable prevalence estimates in comparison to more abundant sequences.

The measured proportion of the 100 most common sequences from the repeated measurements (47 samples taken during 18 runs) on the non-homogeneous clinical sample also varied by run (Fig. 3A). Observed variability was inversely correlated with sequence prevalence (ie. measurements became more variable with in rare sequences). The overall median coefficient of variation (CV) was 36.6% (IQR 34.7–39.3) in the forward direction and 37.9% (IQR 32.1–42.6) in the reverse direction for more abundant sequences (≥1% prevalence) compared to 70.6% (IQR 57.2–92.4) in the forward direction and 75.0% (IQR 64.1–95.9) in the reverse direction for minority species below 1% prevalence (Fig. 3A). This suggests that very low prevalence variants may not be reproducibly measured by the assay. A similar trend was observed by comparing reads in the forward and reverse direction in the 1521 samples with deep sequencing results from the MOTIVATE/A4001029 study. This showed sequences with less than 1% prevalence had a median CV of 27.2% (IQR 12.7–46.0), while sequences with 1% or greater prevalence had a coefficient of variation of 9.9% (IQR 3.9–22.1) (Fig. 3B).

### The effects of input copy number

**Figure 4:**
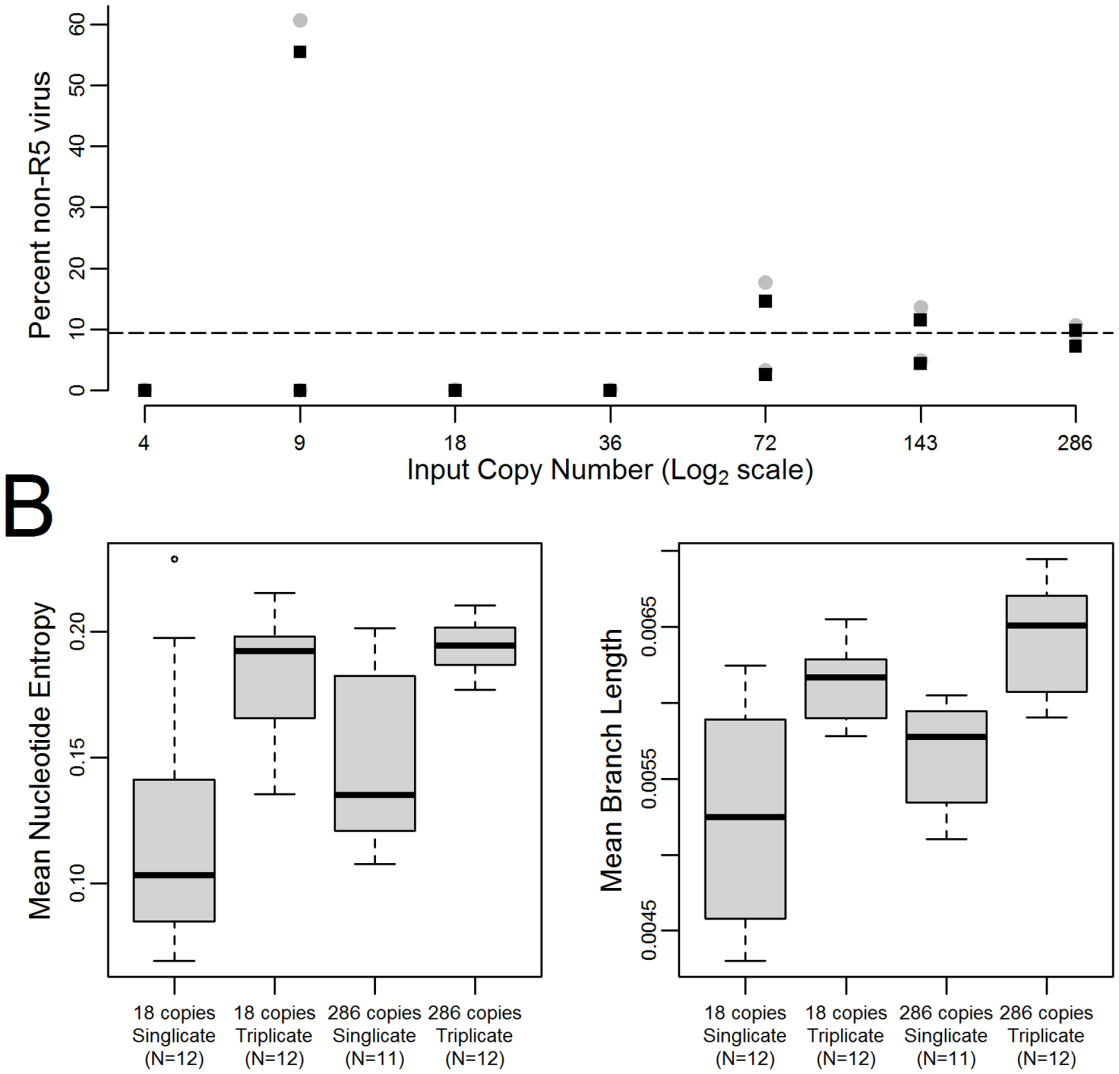
Sequence differences by viral load and PCR replication. A) Percent non-R5 virus detected at varying input copy numbers for a non-homogeneous clinical sample. The dashed line represents the approximate true value for percent of non-R5 virus. B) Measures of viral diversity for 18 and 286 input copies on the non-homogeneous clinical sample amplified with a single PCR and with triplicate PCR. Nucleotide entropies are shown on the left, mean phylogenetic branch length is shown on the right. Estimates tended to be more accurate when the input copy numbers were higher.

In order to test the effect of viral load on sequence measurement, the non-homogeneous clinical sample was sequenced at various viral loads. The measured proportion of non-R5 virus was significantly affected by viral load (Fig. 4A; Wilcoxon rank sum test between viral load 8580 copies/mL and 536 copies/mL p = 0.039), with increasing variability in the measurements at lower viral loads, particularly viral loads below 2000 copies/mL (Fig. 4A). Levene's test for unequal variance showed a significant decrease in variance between 18 and 286 input template copies/reaction for either single repeats or triplicates (p<<0.001 and 0.02 for single replicates and triplicates respectively). Increasing the replicates at a constant viral load stratum significantly decreased the variability of measured non-R5 prevalence at 18 input template copies per reaction (Levene's test p<0.001), but this effect was not significant at 286 input template copies per reaction (p = 0.08).

Sequence diversity increased both with increasing viral load and number of replicates (Fig. 4B). The increase in sequence diversity with increasing viral load was significant (Wilcoxon rank sum test, p<0.001) by all measures including nucleotide entropy, amino acid entropy, phylogenetic tree branch number, phylogenetic tree branch length, and overall phylogenetic tree length. Increasing replicates significantly increased measured sequence diversity in average tree length, and average number of branches (Wilcoxon rank sum test, p values<0.001) but not in the other measures (Wilcoxon rank sum test, p values from 0.14–0.30). The observed increases in measured sequence diversity and decreases in the variability of measured proportion non-R5 virus likely represent a reduction in PCR selection bias with increasing viral load and replication by increasing the initial number of viruses sampled.

### Quality Control Beads

Control bead accuracy differed by bead-specific sequence (Kruskal-Wallis rank sum test, p<<0.001), though the overall average accuracy was still >99% regardless sequence (Range = 99.7–99.9%). Average control bead accuracies also differed over time with some periods having more low quality reads than others (Fig. S2). No significant differences in accuracy between the four physically separated regions of the GS-FLX plate were observed (Kruskal-Wallis rank sum test, p = 0.6073). Proportion of ≤95% accuracy reads also differed by bead, where beads with fewer homopolymer tracks of 4 or more, TF2LonG, TF7LonG, and TF90LonG (2, 3, and 3 homopolymer tracks) had similar proportions of reads with 95% accuracy or less (0.088%, 0.078%, and 0.087% respectively), while Tf150MMP7A, TF100LonG and TF120LonG (7, 6, and 5 homopolymer tracks) had a higher proportion of lower quality reads (0.12%, 0.20%, and 0.34% respectively).

**Figure 5:**
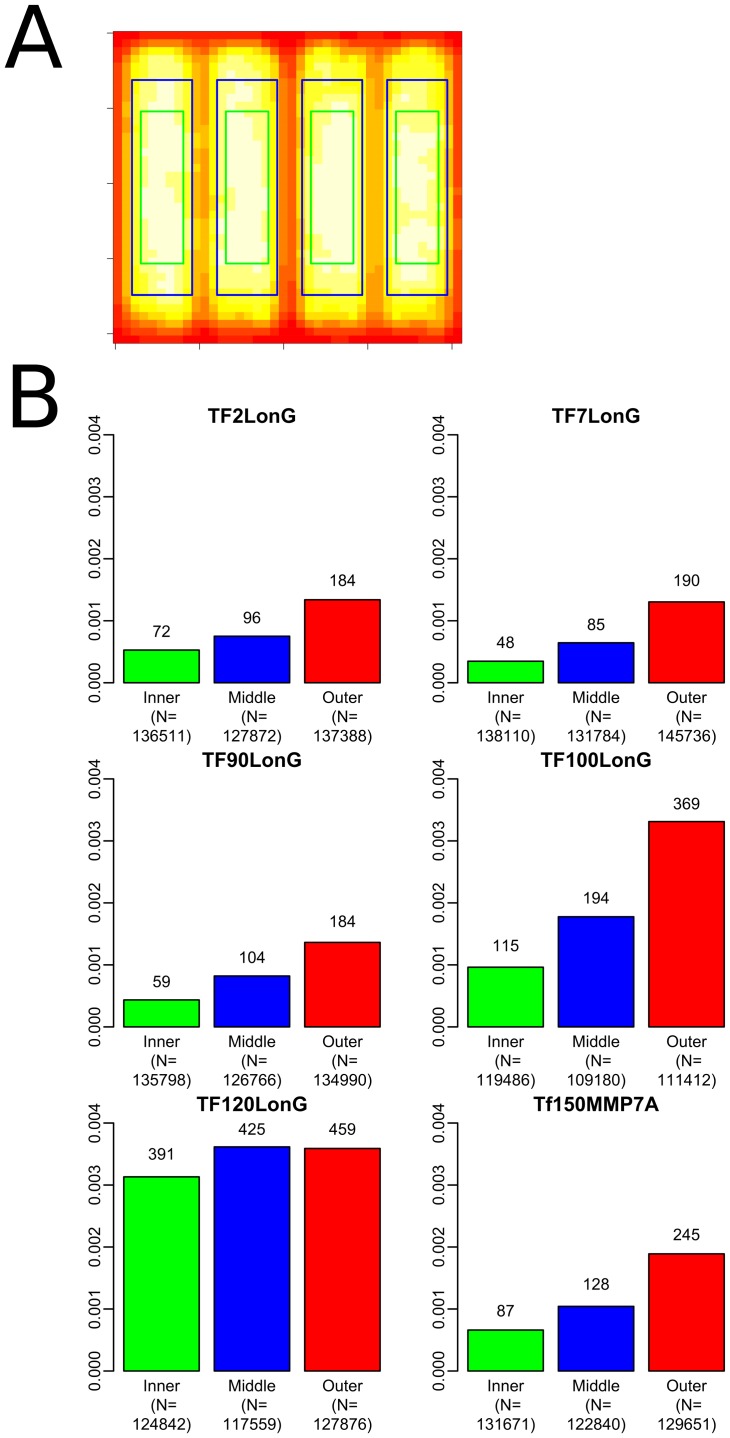
Spatial distribution of control bead accuracy. A)The density distributions for 111 runs of a single control bead (TF2LonG) are shown as an example plate with lighter colours representing higher read density. Regular areas of low density show areas covered by the gasket which physically separates sequencing regions. The division of reads for later analyses are shown as coloured boxes, with reads inside the bluegreenblue box being counted in the inner area, reads between the blue and green boxes as in the middle area, and anything outside of the blue box as being from the outer area. B) Proportion of reads with 95% or lower accuracy by area on the **plate** are shown for each bead in the inner (green), middle (blue) and outer (red) areas respectively. Proportion in percent is shown on the y axis. The total number of reads with an accuracy less than or equal to 95% are shown above each bar while the total number of reads in the region are shown underneath the bar. In general, the number of reads with an accuracy less than or equal to 95% increases towards the outer regions. TF120LonG however, was an exception, having no difference between the middle and outer areas (both with a prevalence of 0.0036%), though the middle and outer areas followed the trend of having more lower accuracy reads than the inner area.

In general, reads were distributed most densely in the center of each region and were less dense toward the edges. Lower accuracy reads occurred most frequently in areas with lower read density. In order to test whether low accuracy reads were in fact more likely to occur towards the edge of a region we divided the overall area of the plate into inner, middle and outer areas with the inner and middle areas each consisting of one quarter of the total area, and the outer area consisting of the remaining half (Fig. 5). This resulted in an approximately equal number of total reads in each area for each bead. Significant differences were present in the read accuracy between areas, with both the inner and middle areas having consistently lower numbers of reads with 95% accuracy or below compared to the outer area (p<<0.001; Fig. 5). The increasing proportions of lower accuracy reads towards the outer area of a region was no longer present if 99% accuracy was instead used as a cutoff so this effect is likely limited to the rare, sequence failures rather than a general phenomenon.

### Comparison of Multiplex Identifiers (MIDs)

In order to determine whether the MID used in the second round of PCR could introduce bias, measured non-R5 virus was compared between the different MID-labeled second round PCR primers after amplifying identical first round amplification products from the non-homogeneous clinical sample (N = 47). The amount of non-R5 virus detected by deep sequencing appeared to vary significantly based on the MID (Kruskal-Wallis rank sum test, p = 0.01). The distributions of percent non-R5 virus from each MID, however, appear to overlap substantially (Fig. S3). Importantly, although an effect of MID was observed for our single clinical sample, no such effect of MID was seen in the MOTIVATE/A4001029 dataset (Kruskal-Wallis rank sum test, p = 0.94). Because the MOTIVATE/A4001029 data were considerably more extensive than the data derived from the non-homogeneous clinical sample, we conclude that there was not significant evidence of biased amplification of templates among MIDs.

### Read direction bias

**Figure 6:**
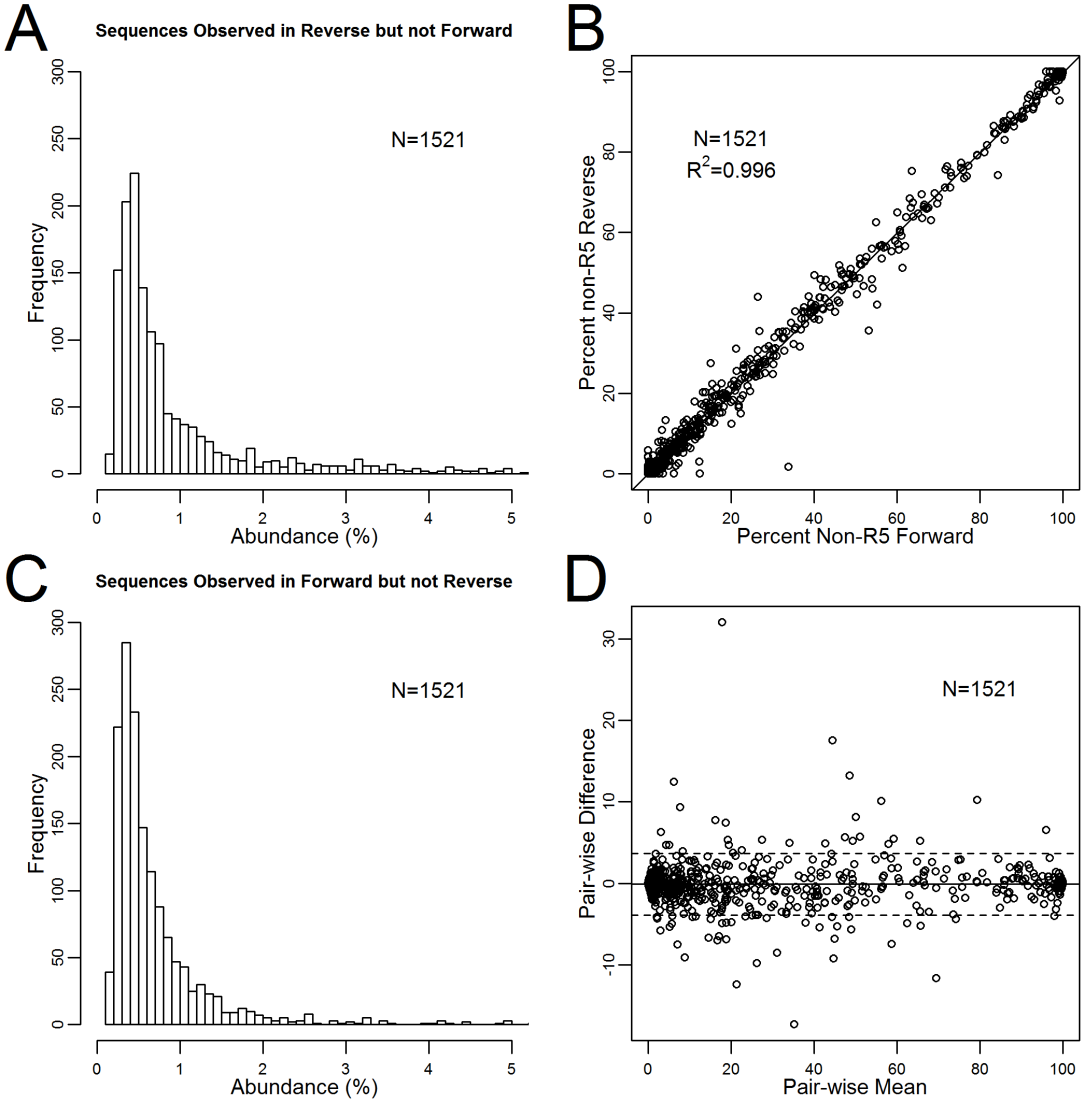
Sequence reproducibility by read direction. A) Histograms of the most common sequence detected in the reverse direction but missing in the forward direction for the MOTIVATE/A4001029 dataset (N = 1521). Less abundant sequences were more likely to be detected only in a single direction compared to sequences with higher abundance. B) Correlation of measured proportion non-R5 for the MOTIVATE/A4001029 dataset between the forward and reverse direction (N = 1521). A linear regression is shown as a solid line. C) Histograms of the most common sequence detected in the forward direction but missing in the reverse direction for the MOTIVATE/A4001029 dataset (N = 1521). D) The lower panel contains a Bland-Altman comparison. Mean difference is plotted as a solid black line and limits of agreement are shown as dotted lines.

Sequences generated in the forward and reverse direction for the MOTIVATE/A4001029 study data tended to agree even for low prevalence sequences. The median prevalence of sequences detected in the forward direction but not the reverse direction was 0.492% (IQR = 0.341–0.819%) (Fig. 6A). The median prevalence of sequence detected in the reverse direction but not the forward direction was 0.621% (IQR = 0.404–1.39%) (Fig. 6A). While the difference was statistically significant (Wilcoxon signed rank test with continuity correction p<<0.001), sequencing in the forward and reverse directions were equally able to detect the prevalence of non-R5 virus (Fig. 6B).

## Discussion

We performed a large scale analysis of the reproducibility and accuracy of 454-based deep sequencing as applied to tropism prediction in HIV and identified a number of factors which can influence these. This was done via an analysis of sequencing results from a number of sources including patient samples, laboratory grown viruses of known tropism, and known control bead sequences. Further, deep sequencing results for the detection of non-R5 virus were compared to other testing strategies; Sanger sequencing and the Trofile assay for sequence result and tropism result respectively.

Consistent with results previously obtained for population-based sequencing [20], both input copy number and PCR replication were key in the detection of minority species as well as the variability in this detection. Detection of minority non-R5 virus, for example, showed discordant results between phenotypic and sequence assays primarily at low non-R5 virus prevalence. Here we additionally show that input template copy number and replication have strong effects on overall sample diversity, where samples with low input copy number show less diversity than samples with higher input copy number, though increasing PCR replication (and thus sampling) can help compensate this effect. This implies that as suggested in [20] amplification steps represent a Poisson sampling which can introduce error. This also helps to explain the observed increased in variability in low prevalence minority species, as these particular minority species would be less likely to appear in any given sampling. This observation is consistent with observations of increased variability in low expression transcripts shown in both RNA-seq data [25] and deepCAGE data [26]. The primer ID method could have allowed input template copy quantification the accurate identification of PCR introduced error [27], and thus have improved the current study. This method was not, however, available at the time of this study. In addition, the primer ID approach would not necessarily be able to correct for the effects of low input copy number, which is increasingly common in treated HIV infected individuals – but it could be used to identify cases with very low input copy numbers The present data suggest that either high input copy number, or multiple replicates should be used to increase sample size and thus minimize sampling bias and/or chance variation.

A number of additional sources of error were also observed including sequence intrinsic factors, temporal factors, and read-position related errors. As the issue of errors resulting from homopolymers has been well documented, it is not surprising that the sequence can influence the read accuracy [1]. Variation of accuracy over time likely represents a mix of random and systematic errors in PCR and sequencing reactions. Biases introduced by MID could not be ruled out, however, these did not appear to have a substantial effect in the large clinical MOTIVATE/A4001029 dataset suggesting that, if present, any biases presented by MIDs is likely not widespread. Perhaps the most unexpected source of error was the effect of read position on accuracy. Lower accuracy reads occurred more frequently towards the edges of a region. This effect may be due to a number of factors including uneven bead deposition or uneven nucleotide flow within a run, both of which may be caused by introducing bubbles at either stage. Since there is an obvious spatial clustering of low accuracy reads, it will be interesting to see if algorithms can be developed on the basis of read location and surrounding control bead accuracy to allow more accurate sequence quality scoring and filtering.

## Conclusions

Deep sequencing appears to be a relatively reliable method for sequence determination and HIV tropism prediction. PCR replication and template availability were important factors in determining the amount of the total population captured by sequencing. A number of sources of error also existed including sequence intrinsic factors, run-specific differences, and position-related read errors. Despite these errors, however, deep sequencing was able to detect the presence of non-R5 virus when these viruses comprised at least 1% of the total population, though the variability in this detection increased dramatically below the 1% threshold.

## Supporting Information

Figure S1 **Difference between measured and known proportion of non-R5 virus.** A direct comparison between the proportion non-R5 virus measured by deep sequencing and known proportion non-R5 (%NL4-3 in Bal) is shown in the upper panel. A linear regression is shown as a solid line. Residuals of the observed proportion non-R5 compared to the known proportion non-R5 are shown in the lower panel. Sequences in the forward direction are shown in black, sequences in the reverse direction are shown in grey. 95% confidence intervals are shown as dotted lines.
(TIFF)

Figure S2 **Control bead accuracy over time.** Accuracy is shown on the Y axis. X axis show run ordered based on date. TF100LonG is shown in green, TF120LonG is shown in grey, Tf150MMP7A is shown in brown, TF2LonG is shown in black, TF7LonG is shown in red, and TF90LonG is shown in blue.
(TIF)

Figure S3 **Percent non-R5 measurements by MID.** Percent non-R5 virus stratified by MID is shown for replicates of the repeated clinical sample derived from the same first round amplicon (N = 47). Percent non-R5 measured in the forward direction is shown in black, and in the reverse direction in grey.
(TIFF)

Methods S1 (DOCX)
