# Supplementary figures and images for: “Deep” Sequencing Accuracy and Reproducibility Using Roche/454 Technology for Inferring Co-Receptor Usage in HIV-1
Source: PLoS One. 2014 Jun 24;9(6):e99508. doi: 10.1371/journal.pone.0099508 (PMC4069016; doi:10.1371/journal.pone.0099508)

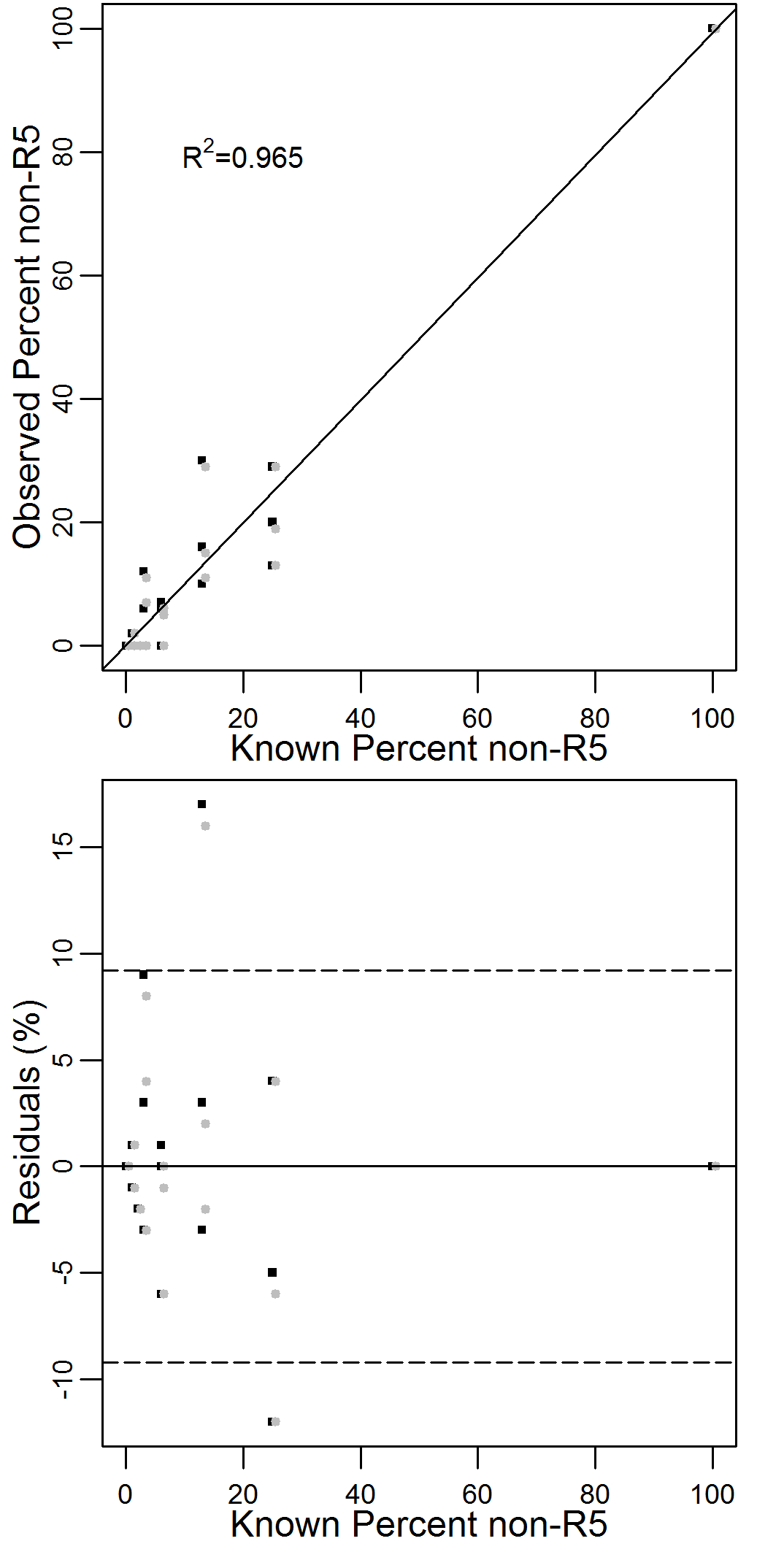

Supplement: Figure S1 — Difference between measured and known proportion of non-R5 virus. A direct comparison between the proportion non-R5 virus measured by deep sequencing and known proportion non-R5 (%NL4-3 in Bal) is shown in the upper panel. A linear regression is shown as a solid line. Residuals of the observed proportion non-R5 compared to the known proportion non-R5 are shown in the lower panel. Sequences in the forward direction are shown in black, sequences in the reverse direction are shown in grey. 95% confidence intervals are shown as dotted lines. (TIFF) [file pone.0099508.s001.tiff]

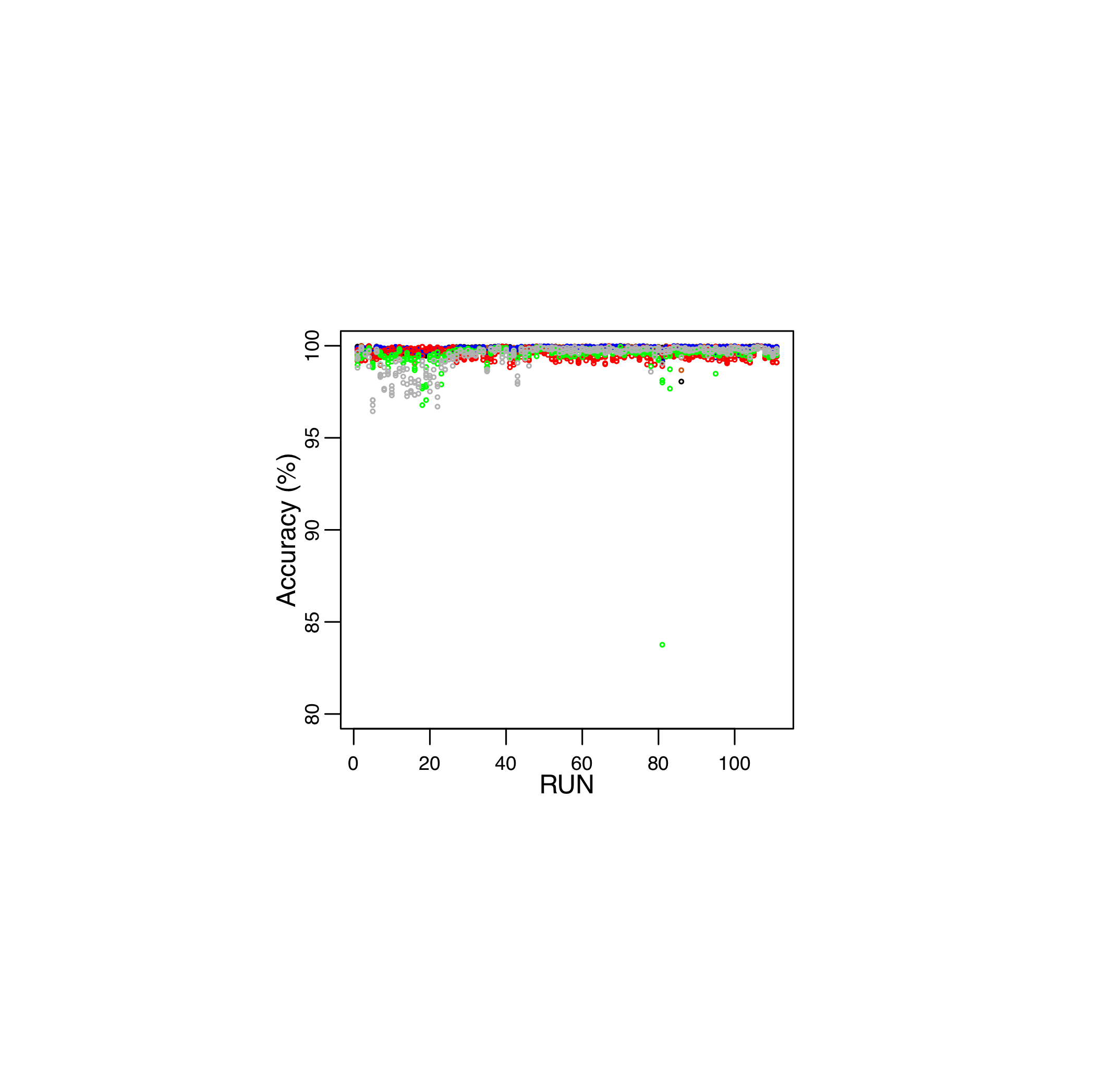

Supplement: Figure S2 — Control bead accuracy over time. Accuracy is shown on the Y axis. X axis show run ordered based on date. TF100LonG is shown in green, TF120LonG is shown in grey, Tf150MMP7A is shown in brown, TF2LonG is shown in black, TF7LonG is shown in red, and TF90LonG is shown in blue. (TIF) [file pone.0099508.s002.tif]

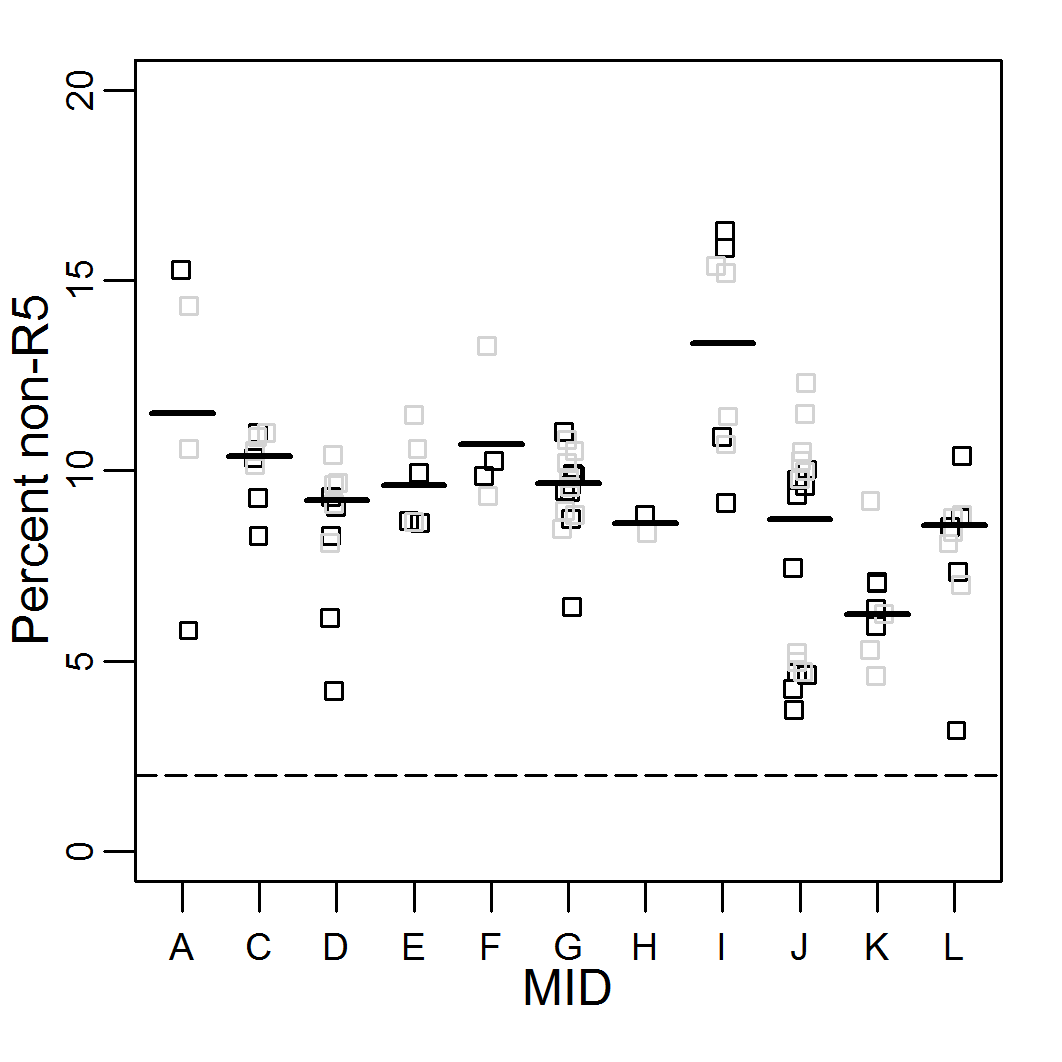

Supplement: Figure S3 — Percent non-R5 measurements by MID. Percent non-R5 virus stratified by MID is shown for replicates of the repeated clinical sample derived from the same first round amplicon (N = 47). Percent non-R5 measured in the forward direction is shown in black, and in the reverse direction in grey. (TIFF) [file pone.0099508.s003.tiff]
