# Supplementary material for: “Deep” Sequencing Accuracy and Reproducibility Using Roche/454 Technology for Inferring Co-Receptor Usage in HIV-1
Source: PLoS One. 2014 Jun 24;9(6):e99508. doi: 10.1371/journal.pone.0099508 (PMC4069016; doi:10.1371/journal.pone.0099508)
Supplement: Methods S1 — (DOCX) [file pone.0099508.s004.docx]

**Supplementary Methods**

*One Step RT-PCR*

Each sample is subjected to reverse transcription followed by PCR using the Invitrogen SuperScript™ III One-Step RT-PCR System with Platinum**^®^** High Fidelty *Taq* Polymerase. For this, a total of 4 µL of extracted RNA are added to a mix containing 10.56 µL DEPC-H_2_O, 4 µL 50% Sucrose solution with 0.04% Bromophenol Blue, 20 µL 2X Reaction Mix (containing 0.4 mM each dNTP and 2.4 mM MgSO_4_), 0.32 µL 25 µM Forwards Primer SQV_3_F_1_ (5' GAG CCA ATT CCC ATA CAT TAT TGT 3’), 0.32 µL 25 µM Reverse Primer CO602 (5’ GCC CAT AGT GCT TCC TGC TGC TCC CAA GAA CC 3’), and 0.80 uL SuperScript™ III RT/ Platinum**^®^** *Taq* High Fidelty Enzyme Mix. The reaction was performed on a thermocycler with the program as follows:

30 minutes at 52º C, 2 minutes at 94º C, 40 cycles of [15 sec. at 94º C, 30 sec. at 55º C, 1 min. 30 sec. at 68º C], and finally 5 minutes at 68º C.

*Nested PCR*

A second PCR was performed with MID tagged, nested primers for each sample in which 2 µL of amplified product was added to 48 µL of second round PCR mix containing 34.125 µL DEPC-treated purified water, 5.0 µL 60% Sucrose solution with 0.08% Cresol Red, 5.0 µl Expand™ High Fidelity Buffer 2 (10X) with 15 mM MgCl_2_, 2.0 µL 25 mM MgCl_2_, 0.4 µL 25 mM dNTP’s, 0.75 µL 12.5 µM tagged Forwards Primer V3F2 and 12.5 µM tagged Reverse Primer V3R, and 0.725 µL of Expand™ HF Enzyme Mix (3.5U/µl). The reaction was performed on a thermocycler with the program as follows: 2 minutes at 94º C, 35 cycles of [15 sec. at 94º C, 30 sec. at 55º C, 50 sec. at 72º C], 7 minutes at 72º C. Primer pairs were as follows:

MID A

Forwards: 5' GCCTCCCTCGCGCCATCAGACGAGTGCGTAATGCCAAAACCATAATAGTACA 3’

Reverse: 5' GCCTTGCCAGCCCGCTCAGACGAGTGCGTGAAAAATTCCCTTCCACAATTAAA 3’

MID B

Forwards: 5' GCCTCCCTCGCGCCATCAGACGCTCGACAAATGCCAAAACCATAATAGTACA 3’

Reverse: 5' GCCTTGCCAGCCCGCTCAGACGCTCGACAGAAAAATTCCCTTCCACAATTAAA 3’

MID C

Forwards: 5' GCCTCCCTCGCGCCATCAGAGACGCACTCAATGCCAAAACCATAATAGTACA 3’

Reverse: 5' GCCTTGCCAGCCCGCTCAGAGACGCACTCGAAAAATTCCCTTCCACAATTAAA 3’

MID D

Forwards: 5' GCCTCCCTCGCGCCATCAGAGCACTGTAGAATGCCAAAACCATAATAGTACA 3’

Reverse: 5' GCCTTGCCAGCCCGCTCAGAGCACTGTAGGAAAAATTCCCTTCCACAATTAAA 3’

MID E

Forwards: 5' GCCTCCCTCGCGCCATCAGATCAGACACGAATGCCAAAACCATAATAGTACA 3’

Reverse: 5' GCCTTGCCAGCCCGCTCAGATCAGACACGGAAAAATTCCCTTCCACAATTAAA 3’

MID F

Forwards: 5' GCCTCCCTCGCGCCATCAGCGTGTCTCTAAATGCCAAAACCATAATAGTACA 3’

Reverse: 5' GCCTTGCCAGCCCGCTCAGCGTGTCTCTAGAAAAATTCCCTTCCACAATTAAA 3’

MID G

Forwards: 5' GCCTCCCTCGCGCCATCAGCTCGCGTGTCAATGCCAAAACCATAATAGTACA 3’

Reverse: 5' GCCTTGCCAGCCCGCTCAGCTCGCGTGTCGAAAAATTCCCTTCCACAATTAAA 3’

MID H

Forwards: 5' GCCTCCCTCGCGCCATCAGTAGTATCAGCAATGCCAAAACCATAATAGTACA 3’

Reverse: 5' GCCTTGCCAGCCCGCTCAGTAGTATCAGCGAAAAATTCCCTTCCACAATTAAA 3’

MID I

Forwards: 5' GCCTCCCTCGCGCCATCAGTCTCTATGCGAATGCCAAAACCATAATAGTACA 3’

Reverse: 5' GCCTTGCCAGCCCGCTCAGTCTCTATGCGGAAAAATTCCCTTCCACAATTAAA 3’

MID J

Forwards: 5' GCCTCCCTCGCGCCATCAGTGATACGTCTAATGCCAAAACCATAATAGTACA 3’

Reverse: 5' GCCTTGCCAGCCCGCTCAGTGATACGTCTGAAAAATTCCCTTCCACAATTAAA 3’

MID K

Forwards: 5' GCCTCCCTCGCGCCATCAGTACTGAGCTAAATGCCAAAACCATAATAGTACA 3’

Reverse: 5' GCCTTGCCAGCCCGCTCAGTACTGAGCTAGAAAAATTCCCTTCCACAATTAAA 3’

MID L

Forwards: 5' GCCTCCCTCGCGCCATCAGATATCGCGAGAATGCCAAAACCATAATAGTACA 3’

Reverse: 5' GCCTTGCCAGCCCGCTCAGATATCGCGAGGAAAAATTCCCTTCCACAATTAAA 3’

This reaction gave a 288 base pair amplicon spanning between HXB2 7062 to 7350.

*Sample Quantification*

1 µL of each amplified sample was then quantified using the Invitrogen™ Quant-iT™ PicoGreen® dsDNA Reagent assay on the DTX 880 Multimode Detector (Beckman Coulter, Fullerton, CA, USA). The triplicate amplifications from each sample were then mixed together in equal proportions in order to minimize PCR based sampling error. These were then combined in equal proportions such that each MID was unique to a given sample, allowing 12 samples to be multiplexed in each of the 4 regions (physically separated areas) available on the GS-FLX plate. Samples were then purified using the Agencourt AMPure PCR purification system (Beckman Coulter, Fullerton, CA, USA) and re-quantified as before.

*Deep sequencing*

DNA was added to DNA Capture Beads (Roche, Basel, Switzerland) in a ratio of between 0.6 and 0.7 molecules of amplified product per capture bead. This was then split into 3 separate emulsion PCR (emPCR) reactions for each the forward and reverse directions. EmPCR and DNA bead enrichment were carried out as per the GS FLX amplicon manual (Roche, Basel, Switzerland) except that 3 melt solution washes were performed to elute the DNA beads from the enrichment beads rather than 2, and the final DNA beads were re-suspended in 200 µL of annealing buffer rather than 100 µL. A 10 µL aliquot of enriched beads was then quantified on a Coulter Counter® (Beckman Coulter Fullerton, CA, USA). DNA beads from the forward and reverse directions were combined such that ~250000 beads were present per region. Beads were then run on the GS FLX system (Roche, Basel, Switzerland) as described in the manual.

*Deep Sequence Processing*

First, sets of sample identifier, MID, viral region sequenced, and sequence direction were created for each combination in the sequencing layout. Duplicate sequences in the raw sequence files from the GS FLX were then removed and replaced by a total count of the number of times each sequence was observed. Next, sequences were compared to the MID set for that sequencing run. Any sequences which did not match an MID were discarded. Sequences were then matched to the primer sequences for the run, allowing determination of sequence direction and those with greater than 3 mismatches from the primer sequence were discarded. Sequences in the reverse direction were then replaced by their reverse complement. All remaining sequences were aligned to a V3 standard using a modified Smith-Waterman algorithm (Smith, Waterman, 1981) and sequences with an alignment score of less than 30 (calculated as matches minus mismatches), with sequences where less than 60% of the bases match the alignment, or with a total length (without primer and MID sequence) of 30 bases or less were discarded.

Remaining sequences were then grouped into their respective set based on MID, viral region sequenced, and sequence direction and any set containing less than 20 sequences was discarded. Following this, sequences within a set are compared. Sequences which are identical except for one to two gaps were then merged as these gaps were likely the result of homopolymer issues. Each set was then re-aligned and a consensus sequence taken. All sequences were then aligned to the consensus sequence. Gaps following repeats of 3 or more bases were replaced by the repeated base, and indels were moved to be in frame. Following this, sequences with an alignment score of than 75 or greater (calculated as matches minus mismatches), less than 2 N’s, and for which at least 60% of the bases match the alignment were retained.

Alignments of each set were then merged into a multiple alignment and all sets trimmed to the length of the V3 standard and sequences with a new length of 15 bases of less were discarded. Sequences which were identical within the trimmed length were then merged. Following this, sequences that had single base insertions were removed if they had an overall prevalence of less than 1% percent in a set as these insertions were likely due to homopolymer error. Finally, each set was checked for contamination of common sequences from other sets and contaminating sequences were discarded. In order to screen for potential contamination among samples within a given 454 run, the five most abundant sequence variants were extracted, discarding any with less than 10% prevalence. Next, all reads within a given sample were compared against this reference collection of abundant variants, and rejected if an exact match was found from another sample. The remaining aligned sequences were then used for further analysis.
